# The use of ultrasound to locate a tethered surgical drain: a novel way to achieve fast removal

**DOI:** 10.1186/s12893-020-00929-y

**Published:** 2020-10-31

**Authors:** Hui Li, Yan Du, Jia-bin Wu, Pan Wang, Jun Yang, Ping Hu, Tao Ai

**Affiliations:** 1 Department of Traumatology, Chongqing Emergency Medical Center, Chongqing University Central Hospital, 1 Jiankang Road, Yuzhong District, Chongqing, 400014 China; 2 Department of Ultrasonography, Chongqing Emergency Medical Center, Chongqing University Central Hospital, Chongqing, 400014 China; 3 Department of Anesthesiology, Chongqing Emergency Medical Center, Chongqing University Central Hospital, Chongqing, 400014 China

**Keywords:** Ultrasound, Tethered drain, Sutured, Sliding sign, Complications

## Abstract

**Background:**

It is rare that drains cannot be removed after surgery, however, this situation cannot be completely avoided, and is also hard to deal with. The main reason for a tethered drain is inadvertent suture fixation. At present, no effective way was published or widely accepted to locate the tethered drain.

**Methods:**

Three cases of orthopedic trauma patients experienced unsuccessful removal of the drain after surgery. The ultrasound was used to locate the sutured site of the drain. Based on the sliding sign and vanishing point which can be detected by the ultrasound, the sutured site of the drain can be clearly identified. Finally, the suture was loosened through a small incision, and the drain was completely removed.

**Results:**

The surgical procedure was very successful in all patients. The tethered drain was quickly and completely removed through a small incision with locating by ultrasound. Intravenous antibiotics were administered within 24 h after surgery, and no wound or deep infections occurred.

**Conclusions:**

Ultrasound can be used to locate a tethered drain based on the sliding sign. This method can simplify the release procedure and achieve fast removal of the drain. Furthermore, it will help lower the risk of a retained drain and soft tissue complications.

## Background

Although it is rare that drains cannot be removed after orthopedic surgery, this situation cannot be completely avoided. The main reason for a tethered drain is inadvertent suture fixation during the closure of an incision, and other reasons include the incarceration of soft tissue, local compression, folding, etc. [1, 2]. Several methods can be employed to remove a drain, including continuous traction, rotation, and cutting inside and outside the lumen by using various instruments [3–5]. The above methods have been shown to be partially effective. However, when the fixed point of the drain cannot be determined, the operation may require a long period of time. Repeated attempts can also damage the drain and soft tissues [6]. As a result, the risk of wound infection is increased. Reopening the incision and extricating the drain require the patient to return to the operating room and may lead to medical disputes, but this is also the safest and most effective method. If the incision is large, searching for the sutured site without a good plan will cause unnecessary damage to the soft tissue. Locating the sutured point of the tube by experience is often unreliable. We report a method using ultrasound to quickly locate a tethered drain based on indirect visualization. This method may help remove the drain more quickly and safely.

## Methods

Last year, 3 patients experienced unsuccessful removal of the drain after surgery. These patients all received orthopedic surgeries, includes femoral intramedullary nailing fixation, pedicle screw fixation for the second lumbar vertebra, and open reduction and internal fixation for distal femoral fractures. The silicone drains were placed at the time of the procedure without the drain being secured with a fascial closure. Attempts were made to remove it on the second or third postoperative day, which was met with great resistance. Considering that the drains were likely to be sewn by mistake, no more attempts were made. Finally, we decided to remove the drains by open the incisions limitedly under anesthesia.

The first patient was transferred to the operating room. Because of the long incision, it was difficult to choose where to reopen the incision. When the anesthesiologist was preparing for ultrasound-assisted nerve block anesthesia, we discussed trying to locate the sutured site of the drain by ultrasound. Finally, we used ultrasound to clearly identify the route of the drain. By slowly pulled and relaxed the drain several times, the sliding sign can be detected by the ultrasound, which indicates the point where the drain was sutured. Finally, the suture was loosened through a small incision, and the drain was completely removed. The second and third patients were not transferred to the operating room, the sutured drains were removed successfully through small incisions positioned by ultrasound. To better characterize the image of the drain under ultrasound and the imaging features of the sliding sign, we demonstrated the process of removing drain based on the pork model.

**Fig. 1:**
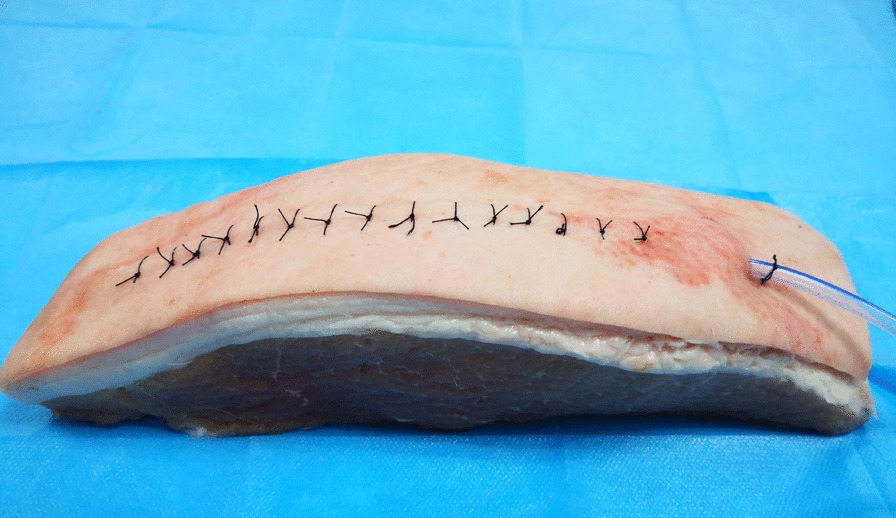
The operation process was simulated by using pork phantoms. The drain was sutured at the segment located approximately 3 cm from the proximal end and could not be removed smoothly

**Fig. 2:**
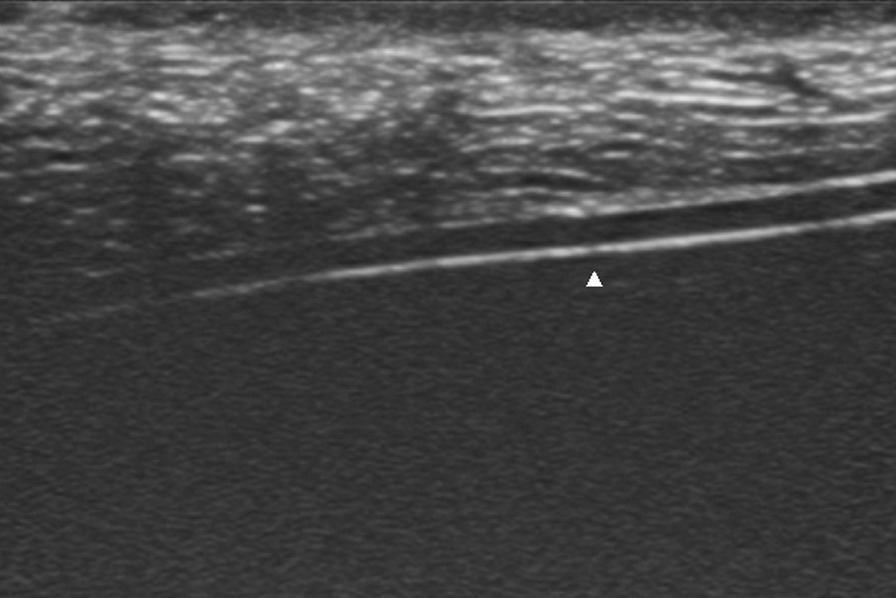
Longitudinal view of the tube. The anterior and posterior walls of the tube are clearly indicated, and the side hole is represented by the white triangle. Pulling and relaxing the drain repeatedly can cause a sliding sign

**Fig. 3:**
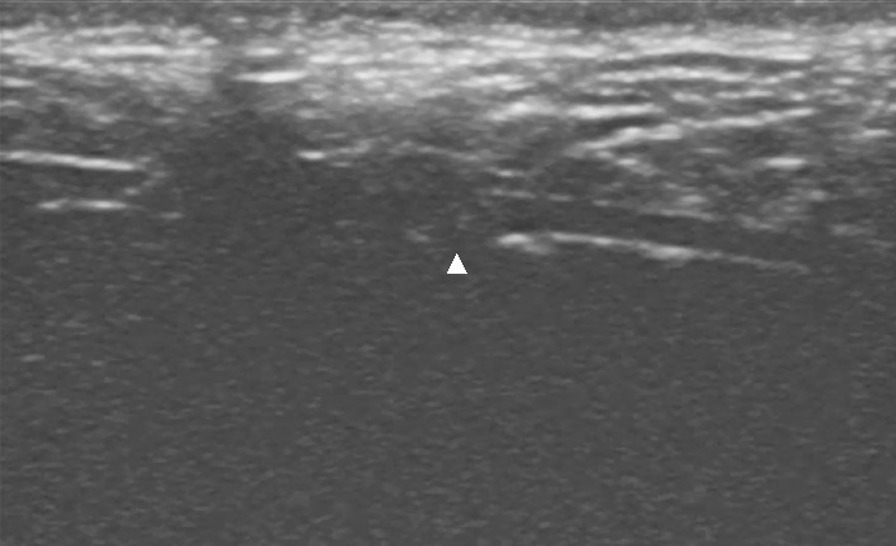
The site where the sliding sign disappears (vanishing point), shown by a white triangle, is where the drain is tethered. The motionless proximal end is shown in the scan

**Fig. 4:**
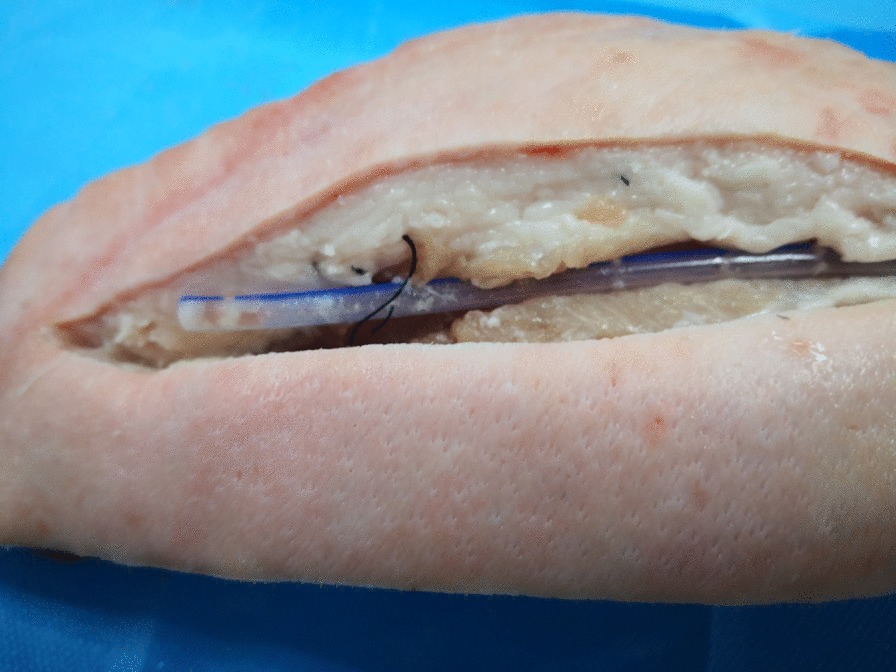
The sutured site of the drain located by ultrasound was confirmed by reopening the incision

Pork models were used because they are simple and cost-effective models and have similar anatomic structures and echogenicities as human tissue. A boneless pork phantom (28 × 8 × 6 cm) was used. We simulated the operation at room temperature (23 °C). The drain (XY-16Fr), which was produced by the Chinese company XIANGYUE, was inserted into the phantom, and the segment approximately 3 cm from the proximal end was sutured. Then, the incision was closed (Fig. 1). The tube could not be removed after repeated traction. An ultrasound machine (Mindray, M9) equipped with a high-resolution 7–10 megahertz (MHz) linear array transducer was used. First, the drain was scanned transversely to identify the cross-section, and then the skin was marked to determine the length and path of the drain. Afterwards, the longitudinal view of the drain, the front and rear wall, and the side hole of the drain were detected (Fig. 2). At this time, the drain was slowly pulled and relaxed repeatedly from the distal end, and it was observed that the drain was sliding in the soft tissue, that is, creating a sliding sign. After the transducer was slowly moved to the proximal end, the segment where the sliding sign disappeared was identified (vanishing point) (Fig. 3). This area is where the drain was being tethered. A video of this process from the ultrasound display is provided in the Additional files 1 and 2 (Video S1 and Video S2). The tube segment located by ultrasound was consistent with the sutured site (Fig. 4). With the use of ultrasound, a targeted incision was made, and the tethered drain was removed quickly without unnecessary exposure.

## Results

A summary of the demographic and clinical data of the patients is shown in Table 1. All the surgical drains placed in the incisions of the three patients were the same as used in the pork model. The lengths of the drainage tubes under the skin of the three patients were 20 cm, 12 cm, and 20 cm, respectively. The distance between the sutured site and the proximal end of the drain is 3 cm, 4 cm, and 15 cm, respectively. We only loosened the suture site about 2–3 cm and made fast removal. The first patient was operated under nerve block anesthesia and the other two patients received local infiltrating anesthesia.

**Table 1:** Summary of the demographic and clinical data of the patients

	Patient 1	Patient 2	Patient 3
Age	55	31	48
Gender	Male	Female	Male
Diagnosis	Comminuted proximal fracture of the right femur	The second lumbar vertebra fracture	Distal femoral fracture
Surgery	Intramedullary nailing fixation	Pedicle screw fixation	Open reduction and internal fixation
Length of drain	20 cm	12 cm	20 cm
Sutured site from the proximal end	3 cm	4 cm	15 cm
operation time	15 min	5 min	5 min
Length of the incisions reopened	3 cm	2 cm	2 cm
Complications	No	No	No
Follow-up	10 months	6 months	2 months

The operation time in the first patient was 15 min, and about 5 min in the second and third patient. The surgical procedure only needs a short time, most of the time in the process was spent on locating the tethered drain by ultrasound. The drains were completely removed, and the cut marks also can be found. The tethered site of the drains located by the ultrasound was confirmed after the operation. All patients received intravenous antibiotics within 24 h after surgery, and no wound infection occurred. So far, the three patients have been followed up for 10 months, 6 months, and 2 months, respectively, and no deep infection involving internal fixator has been observed.

## Discussion

At present, there are several techniques available to prevent drains from being tethered during operations [7, 8]. It is mainly important to ensure that the drain is slack and can be pulled before the incision is closed. However, it is not uncommon that drains cannot be removed smoothly for various reasons. The main reason for the inability to remove a drain is the inadvertent placement of a suture during the closure, which is especially common in orthopedic operations [2]. According to our surgical experience, it is estimated that the incidence of tethered drains is between 0.1 and 0.5%.

Ultrasound can be used to locate the tethered site of a drain quickly and effectively. To the best of our knowledge, this is the first report describing this use of ultrasound for this purpose. This method is fast and simple to use, and the skills can be quickly mastered by the novice without ultrasound experience. Several minimally invasive methods for removing drains have been reported in the literature, but little attention has been paid to how to locate a tethered drain. When the tethered site is not cleared, repeated attempts can cause damage to the drain and increase the risk of soft tissue injury and infection [6].

Ultrasound scanning can be used to locate the position of the drain accurately, visualize the operation process, shorten the operation time, reduce tissue damage, and lower the risk of infection. When a drain is tethered, the main goal is to ensure complete removal [6]. Keeping the operation minimally invasive and the incision intact is the secondary goal. The significance of an ultrasonic evaluation is to visualize the management process and ensure that the drain is completely and smoothly removed. Theoretically, the loosened process of the drain can be simplified when the tethered site is cleared, regardless of whether the minimally invasive procedure is performed inside or outside of the lumen. If the drain is removed by constant traction or rotation [9], ultrasound can also be used for real-time evaluation. Finally, even if the drain is damaged, the previous positioning also helps clinicians quickly identify the remaining debris. If there is more than one suture in the incision, after one suture is removed, the left sutured site can also be detected by ultrasound.

Under ultrasound scanning, the sliding sign can be easily created by pulling the distal end of the drain. However, during traction, the proximal tube is still motionless. The junction point of movement and rest with the tube can be easily observed with ultrasound. The tethered drain can be quickly located in the junction segment. The sliding sign is obvious on an elastic drain, for instance, a silicone tube. If the drain is inelastic, we have found that the sliding sign can be detected by rotating the distal end of the drain to successfully locate the tethered part. The side holes on the drain can also be clearly identified under ultrasound [10]. In ultrasound scanning, these side holes can help clinicians locate the sliding signs and assist the tethered drain.

## Conclusion

Here, we report the application of ultrasound in locating a tethered drain. This method can simplify operations to release sutures. Decisions can be made based on the sliding sign, and the operation can be visualized, which promotes the clinical use of this technology. We recommend that when a drain is suspected to be inadvertently sutured, the first step is to use ultrasound to accurately locate the tethered site. Whether the drainage tube is removed by minimally invasive methods or the incision is reopened in the theater, identifying the tethered site of the drain beforehand will simplify the operation process. Furthermore, it will help lower the risk of a retained drain and local tissue complications.

## Supplementary information

**Additional file 1: Video S1.** Creating a sliding sign. Pulled and relaxed the drain repeatedly from the distal end, and the drain can be observed in the soft tissue by the ultrasound.

**Additional file 2: Video S2.** Vanishing point. Move the transducer slowly moved to the proximal end, the segment where the sliding sign disappeared was identified, this is the point where the drain was tethered.

## Data Availability

The datasets used during the current study available from the corresponding author on reasonable request.

## Abbreviation

MHZ: Megahertz
